# The value of arthroplasty registries: from surveillance to societal impact

**DOI:** 10.2340/17453674.2026.46171

**Published:** 2026-05-29

**Authors:** Ola ROLFSON, John McKIE, Anne LÜBBEKE

**Affiliations:** 1 Institute of Clinical Sciences, Sahlgrenska Academy, University of Gothenburg; 2 Sahlgrenska University Hospital, Gothenburg; 3 Swedish Arthroplasty Register, Gothenburg, Sweden; 4 New Zealand Joint Registry, Christchurch, New Zealand; 5 Division of Orthopaedic Surgery and Musculoskeletal Trauma Care, Geneva University Hospitals, Geneva; 6 Faculty of Medicine, University of Geneva, Geneva, Switzerland; 7 Nuffield Department of Orthopaedics, Rheumatology and Musculoskeletal Sciences, University of Oxford, UK

## Abstract

**Info** — Since early 2025, *Acta Orthopaedica* has served as the official publisher of the International Society of Arthroplasty Registries (ISAR) congresses. Founded in 2005, ISAR (https://www.isarhome.org) is a global organization comprising national, regional, and institutional joint replacement registries. Its mission is to improve outcomes for patients undergoing joint replacement surgery worldwide.

Since 2012, ISAR has organized annual congresses featuring register-based research. Following the 14th International Congress of Arthroplasty Registries, held in Christchurch, New Zealand, presenters were invited to submit their work to *Acta Orthopaedica*. Over the past year, this initiative has resulted in 13 publications covering a broad range of topics. This partnership has been extended, looking ahead to the 15th congress held in Lund May 30 to June 1, 2026.

Presenters at the Lund Congress are welcomed to submit their studies to *Acta Orthopaedic* with a deadline of October 1, 2026.

Arthroplasty registries have become a cornerstone of modern orthopedic practice. Over the past 5 decades, they have evolved from surveillance tools into complex infrastructures that inform clinical decision-making, support regulatory oversight, and drive quality improvement at scale. Yet, in an era of constrained healthcare resources, a fundamental question persists: do arthroplasty registries provide value for money?

Recent economic evaluations of the Dutch Arthroplasty Register and the Australian Orthopaedic Association National Joint Replacement Registry (AOANJRR) provide important insights [1,2]. Together with the breadth of contemporary register-based studies [3-15] presented at the 14th International Congress of Arthroplasty Registries in Christchurch, they reinforce a compelling conclusion: registries are indispensable, not only clinically, but economically and societally.

## Small improvements may yield large returns

The Dutch evaluation demonstrates that preventing as few as 1 to 2 revisions per hospital over decades is sufficient to offset registry costs [1]. Even marginal improvements in outcomes yield substantial value at population level.

The Australian evaluation strengthens this message. The AOANJRR proved economically dominant, improving patient outcomes while reducing overall healthcare costs, with a benefit–cost ratio exceeding 10:1 [2]. Importantly, these estimates include only a subset of measurable benefits, suggesting that the true value of registries is likely far greater.

## Registries as engines of quality improvement

Registries generate value primarily through their capacity to identify variation and enable change. Benchmarking, feedback, and outlier detection create actionable insights that translate into improved care. The Dutch data suggest that focusing on poorly performing hospitals alone could surpass thresholds for cost-effectiveness.

Crucially, registries also provide the infrastructure necessary to evaluate improvement strategies. Through their continuous monitoring activity, they demonstrate iterative learning cycles across entire healthcare systems, which is unattainable with traditional study designs.

## A broad evidence ecosystem: insights from the Christchurch meeting

The studies presented in Christchurch collectively demonstrate that registry value extends well beyond revision rates, covering implants, techniques, health systems, and patient-reported outcome measures (PROMs). Thanks to the partnership between ISAR and Acta Orthopaedica, 13 of these studies have been published open access in the journal over the last year.

## Implant surveillance and material science

Register-based monitoring remains central to patient safety and innovation. An Australian study of nearly 200,000 hip replacements showed a reduced revision risk with antioxidant-added highly cross-linked polyethylene, providing large-scale evidence to guide implant selection [3]. Similarly, registry analyses continue to identify performance differences between fixation methods, bearings, and implant designs, facilitating rapid translation into practice.

In parallel, Canadian data on fixation in hip fracture arthroplasty demonstrated evolving trends in the use of cemented implants and associated predictors, reflecting how registries capture shifts in real-world practice over time [4].

## Surgical technique and perioperative practice

Large-scale register datasets uniquely enable evaluation of surgical decisions at a population level. Norwegian data on more than 300,000 procedures addressed optimal antibiotic prophylaxis strategies, directly informing infection prevention [5]. Swedish analyses of tourniquet use in total knee replacement found no clear benefit regarding revision risk, supporting a reconsideration of routine practice [6].

Together, these studies highlight how registries provide pragmatic, high-powered evidence where randomized trials are often infeasible or even impossible.

## Health services research and system organization

A recurring theme from Christchurch is the ability of registries to challenge assumptions regarding healthcare organization. Data from the Netherlands demonstrated that higher hospital volume does not necessarily confer better outcomes in revision total hip replacement, questioning long-held centralization paradigms [7].

Moreover, analyses of over 48,000 unicompartmental knee replacements revealed how hospital characteristics influence both revision rates and reasons for failure [8]. These findings illustrate how registries inform policy decisions regarding service delivery, training, and case selection.

Register data also captures real-world variation in practice, including differences in fixation strategies, implant use, and perioperative protocols across jurisdictions.

## PROMs and risk stratification

A major evolution in registries is the integration of PROMs and linked datasets. Danish studies demonstrated that worse preoperative self-rated health is associated with prolonged opioid use after arthroplasty [9], while alternative definitions of opioid exposure significantly influence observed risks and associations with mortality [10].

These findings underscore a critical shift: registries are no longer limited to implants and revisions but increasingly capture the full patient journey, including pain, function, and medication use.

Further, register-based analyses have clarified determinants of patient satisfaction, showing that pain relief and more than functional gains drive perceived success [11]. Such insights are crucial for aligning surgical outcomes with patient expectations.

## Rare conditions, special populations, and emerging risks

Registries are uniquely positioned to study small subgroups and infrequent events. German data on patients aged 30 years or younger undergoing total hip replacement identified important predictors of revision in this high-risk population [12]. Similarly, a US register-based study examined the timing of a COVID-19 infection in relation to total hip and knee replacement and the perioperative risk of thromboembolism and mortality [13].

Linkage to other health data sources has also enabled broader orthopedic insights, such as associations between postoperative mobility and mortality after hip fracture [14], demonstrating the expanding scope of register science beyond elective arthroplasty.

## Exploring variation—even the unexpected

One of the strengths of registries is their ability to explore variability in care, sometimes in unexpected ways. A Dutch study examining “superstition” found no association between dates or implant sizes with the number 13 and revision risk [15]. While lighthearted in concept, such analyses highlight the robustness of registry data in interrogating bias and variation in clinical practice.

## Safety, surveillance, and societal impact

The most visible success of arthroplasty registries remains their role in patient safety. Early detection of poorly performing implants has prevented large-scale harm and enabled timely device withdrawal. The Australian economic evaluation highlights that prosthesis recall contributes significantly to the overall benefit of registries [2].

However, the societal impact extends further. Registries enhance transparency, strengthen trust, and support regulators, clinicians, and patients in making informed decisions. They also reduce downstream costs by preventing complications, reoperations, and litigation. Additionally, they provide valuable contributions to medical education [16].

## The attribution dilemma

A consistent challenge in evaluating registries is disentangling their effects from broader improvements in orthopedic care. Advances in implants, techniques, and perioperative care all contribute to improved outcomes.

Yet this argument overlooks a key point: registries document and thus indirectly enable these advances. They provide the observational knowledge-base and infrastructure necessary to detect trends, validate innovations, and disseminate best practice.

## Looking ahead

The future of arthroplasty registries lies in deeper integration such as linking surgical data with imaging, medical records, primary care, pharmacological, and socioeconomic datasets. Advances in analytics will allow real-time monitoring, predictive modelling, and increasingly personalized care pathways.

At the same time, maintaining high-quality registries requires sustained investment and engagement. The evidence is now clear: registries are not a cost burden but a value-generating component of modern healthcare systems.

## Conclusion

Arthroplasty registries represent one of the most successful examples of data-driven healthcare. Economic evaluations from the Netherlands and Australia demonstrate that even conservative assumptions confirm value for money. The diverse body of research presented at the ISAR congress in Christchurch illustrates their much broader impact, spanning implant safety, surgical practice, healthcare organization, and patient outcomes.

Ultimately, the value of arthroplasty registries lies not only in preventing revisions but in enabling continuous improvement across the entire arthroplasty pathway. Their contribution to safer, more effective, and more sustainable healthcare systems is profound, and will only grow in importance in the years ahead.

## Notes

Complete disclosure of interest forms according to ICMJE are available on the article page, doi: 10.2340/17453674.2026.46171
